# A Turn-On Fluorescent Probe for Sensitive Detection of Cysteine in a Fully Aqueous Environment and in Living Cells

**DOI:** 10.1155/2018/1986468

**Published:** 2018-12-13

**Authors:** Xiaohua Ma, Guoguang Wu, Yuehua Zhao, Zibo Yuan, Yu Zhang, Ning Xia, Mengnan Yang, Lin Liu

**Affiliations:** ^1^School of Chemical Engineering and Technology, China University of Mining and Technology, Xuzhou, Jiangsu 221116, China; ^2^Henan Key Laboratory of Biomolecular Recognition and Sensing, College of Chemistry and Chemical Engineering, Shangqiu Normal University, Shangqiu, Henan 476000, China; ^3^Key Laboratory of New Optoelectronic Functional Materials (Henan Province), College of Chemistry and Chemical Engineering, Anyang Normal University, Anyang, Henan 455000, China

## Abstract

We reported here a turn-on fluorescent probe (**1**) for the detection of cysteine (Cys) by incorporating the recognition unit of 2,4-dinitrobenzenesulfonyl ester (DNBS) to a coumarin derivative. The structure of the obtained probe was confirmed by NMR and HRMS techniques. The probe shows a remarkable fluorescence off-on response (∼52-fold) by the reaction with Cys in 100% aqueous buffer. The sensing mechanism was verified by the HPLC test. Probe **1** also displays high selectivity towards Cys. The detection limit was calculated to be 23 nM. Moreover, cellular experiments demonstrated that the probe is highly biocompatible and can be used for monitoring intracellular Cys.

## 1. Introduction

Cysteine (Cys), a kind of critical biothiols, plays many crucial physiological roles, such as maintaining biological redox homeostasis, participation in enzymatic reactions, and sequestering inimical metal ions [1–4]. The abnormal levels of Cys are associated with many syndromes and diseases, including growth retarding, muscle loss, skin lesions, liver damage, severe neurotoxicity, and cardiovascular diseases [5–7]. Therefore, it is highly desired to develop effective Cys assays for application in biological systems, which would be very helpful to further elucidate its biological functions and reveal its relevance to certain diseases.

Analytical methods for the detection of Cys include capillary electrophoresis (CE) [8–10], highperformance liquid chromatography (HPLC) [11–13], electrochemical methods [14], and colorimetric and fluorescent assays [3, 15–18]. Among them, the fluorescence assay based on optical probes has gained tremendous attentions due to its inherent advantages of high sensitivity and selectivity, simplicity of implementation, high spatiotemporal resolution, and good compatibility for biosamples [19–26]. Up to now, some fluorescent probes have been synthesized for the detection of Cys by exploiting mechanisms of Michael addition, cleavage of the selenium-nitrogen bond and of disulfides, cyclization with aldehydes, cleavage of sulfonamide and sulfonate esters, and metal complex replacement of ligands [27–40]. However, many of these developed probes have drawbacks of low sensitivity, complicated synthetic process, and/or the use of high-content organic solvent. Thus, developing facile and reliable fluorescent Cys probes is still highly desired. Herein, we report a highly sensitive fluorogenic Cys probe (**1**) by installing the recognition moiety of 2,4-dinitrobenzenesulfonyl ester (DNBS) onto a coumarin fluorophore. Coumarin and its derivatives are popular fluorescent reporters due to their high photostability, excellent biocompatibility, and high quantum yield [41–43]. Upon the target-mediated cleavage of 2,4-dinitrobenzenesulfonyl ester and release of the coumarin fluorophore, probe **1** exhibits efficient turn-on fluorescent response towards Cys. Moreover, the proposed probe **1** displays good water solubility, high sensitivity and selectivity, and low cytotoxicity and can be used for imaging intracellular Cys.

## 2. Experimental Section

### 2.1. General Procedure for Analysis

All spectral measurements were performed in the aqueous phosphate buffer (pH 7.4, 10 mM). Stock solution of probe **1** (0.1 mM) was prepared in the same phosphate buffer solution. The following solutions (10.0 mM) were prepared in deionized water: amino acids (Cys, Hcy, GSH, Gly, Ser, Val, Leu, Tyr, His, Trp, Arg, Glu, Pro, Asp, Thr, Asn, and Phe), ascorbic acid (AA), and glutathione (GSH). Test solutions were prepared by placing 300.0 *μ*L of stock solution 1 (0.1 mM), an appropriate aliquot of each analyte stock solution into a 5.0 mL centrifugal tube, and diluting the solution to 3.0 mL with the phosphate buffer (pH 7.4, 10 mM). The solution was mixed for a given time at the room temperature. Then, the fluorescence and UV absorption spectra were recorded. For fluorescence assays, the excitation and emission slit width are both 5 nm.

### 2.2. Synthesis of Probe **1**

**Scheme 1:**
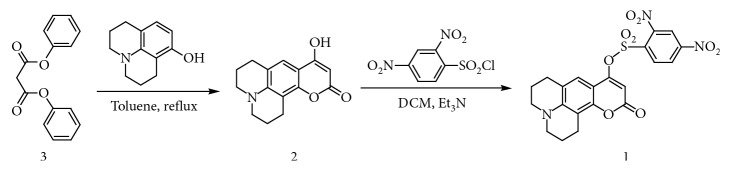
Synthetic route for probe **1**.

Synthesis procedures for probe **1** were displayed in Scheme 1. Compound **3** was obtained according to literature methods [44, 45].

Compound **2**, compound **3** (12.8 g, 50 mmol), 2,3,6,7-tetrahydro-8-hydroxy-1H, and 5H-benz[i, j]quinolizine-9-carboxaldehyde (8.26 g, 50 mmol) were added to toluene (0.1 L), and the mixture was refluxed for 10 h. Then, the formed solid product was filtered and washed with hexanes. The obtained precipitation was further dried under vacuum giving a white solid (8.7 g, 76%). ^1^H NMR (400 MHz, DMSO) *δ*: 11.78 (s, 1H), 7.19 (d, *J* = 31.3 Hz, 1H), 5.22 (s, 1H), 3.23 (s, 4H), 2.70 (s, 4H), and 1.87 (s, 4H) (Figure S1). ^13^C NMR (100 MHz, DMSO): *δ* 167.06 (s), 163.29 (s), 151.46 (s), 146.45 (s), 120.35 (s), 117.78 (s), 105.80 (s), 103.53 (s), 86.25 (s), 49.67 (s), 49.14 (s), 27.42 (s), 21.46 (s), and 20.58 (d, *J* = 2.3 Hz) (Figure S2). HRMS: m/z, calcd. [M + H]^+^ 258.1130; found 258.1126 (Figure S3).

Probe **1** was prepared by reacting compound **2** with 2,4-dinitrobenzenesulfonyl chloride. In brief, compound **2** (2.57 g, 10 mmol), 2,4-dinitrobenzenesulfonyl chloride (2.67 g, 10 mmol), and triethylamine (1.21 g, 12 mmol) were added in anhydrous CH_2_Cl_2_ (0.1 L) at 0°C. After stirring for 1 h, the mixture was gradually warmed to the room temperature and reacted for another 2 h. Then, the reaction mixture was evaporated to dryness and purified by column chromatography (silica, DCM-EtOAc as eluent, 2: 1, v/v) yielded **1** as a yellow solid (12.82 g, 58%). ^1^H NMR (400 Hz, CDCl_3_): *δ* 8.71 (s, 1H), 8.60 (d, *J* = 8.1 Hz, 1H), 8.41 (d, *J* = 8.3 Hz, 1H), 7.16 (s, 1H), 5.87 (s, 1H), 3.30 (s, 4H), 2.79 (d, *J* = 35.0 Hz, 4H), and 1.96 (s, 4H) (Figure S4). ^13^C NMR (101 MHz, CDCl_3_) *δ* 161.98 (s), 158.41 (s), 151.30 (s), 151.19 (s), 148.87 (s), 134.00 (s), 133.36 (s), 126.96 (s), 120.79 (s), 120.06 (s), 119.25 (s), 96.54 (s), 77.34 (s), 77.23 (s), 77.03 (s), 76.71 (s), 50.11 (s), 49.67 (s), 27.54 (s), 21.06 (s), 20.31 (s), and 20.13 (s) (Figure S5). HRMS: m/z, calcd. [M + H]^+^ 488.0764; found 488.0759 (Figure S6).

## 3. Results and Discussion

### 3.1. Design and Synthesis

The probe **1** was devised by exploiting **2** as the fluorophore and DNBS as the reaction moiety. The coumarin derivative (compound **2**) was selected here because of its high emission efficiency, facile preparation procedure, excellent water solubility, and biocompatibility. DNBS group has been exploited as a good reaction moiety for fluorescent biothiols probes. Scheme 1 illustrates the synthesis procedures for probe **1**. Compound **2** was prepared via refluxing malonate ester with 2,3,6,7-tetrahydro-8-hydroxy-1H and 5H-benz[i, j]quinolizine in toluene. Furthermore, coupling **2** with 2,4-dinitrobenzenesulfonyl chloride in CH_2_Cl_2_ afforded **1**. The structures of compound **2** and probe **1** were confirmed by NMR and HRMS (Supporting Information).

### 3.2. Spectral Characteristics of Probe **1** and Its Optical Responses towards Cys

**Figure 1:**
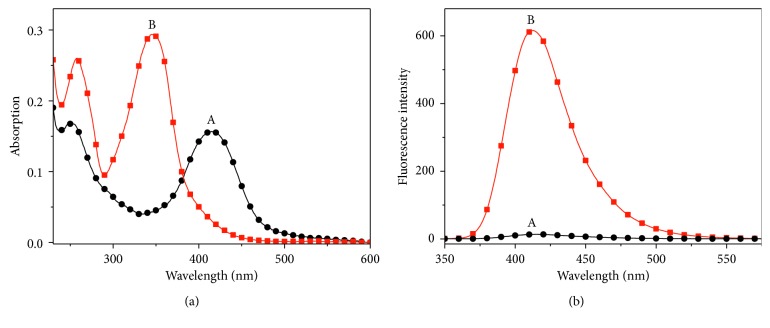
(a) Absorption and (b) emission spectra of probe **1** (10 *μ*M) in the absence (A) and presence (B) of Cys (100 *μ*M) in a solution of the phosphate buffer (pH 7.4, 10 mM). *λ*_ex_ = 347 nm.

The spectroscopic characteristics of probe **1** were inspected with or without Cys (10.0 equiv) (Figure 1). **1** alone displayed an absorption band at about 415 nm (*ε* = 1.57 × 10^4^ M^−1^·cm^−1^) and nonemissivity (curve a). With the addition of Cys (10.0 equiv), the absorbance at 415 nm decreased significantly, and a new absorption band centered at 347 nm (*ε* = 2.93 × 10^4^ M^−1^·cm^−1^) appeared (curve b). Meanwhile, the emission of the probe solution increased remarkably (*λ*_em_ = 413 nm). These obvious spectral responses imply that probe **1** is capable of monitoring Cys.

**Figure 2:**
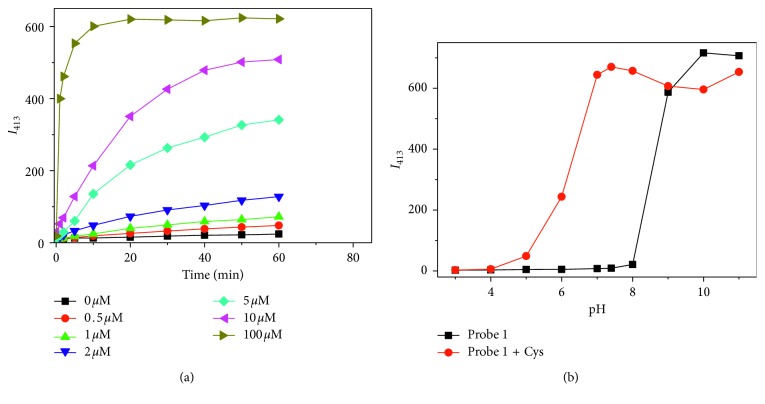
(a) Time-dependent fluorescence intensity changes of probe **1** (10 *μ*M) at 413 nm in the presence of various concentrations of Cys (0, 0.5, 1, 2, 5, 10, and 100 *μ*M); (b) effect of pH on the fluorescence response of probe **1** (10 *μ*M) towards Cys (100 *μ*M). *λ*_ex_ = 347 nm.

To study the response time of probe **1** for Cys, time-dependent fluorescence response of probe **1** towards Cys with different concentrations was investigated (Figure 2(a)). The peak emission intensity of probe **1** did not obviously change in the absence of Cys during the time course of testing, indicating the high stability of the probe in the aqueous buffer solution under the neutral condition. And the emission intensity was observed to increase in the presence of Cys in a concentration-dependent fashion. Higher concentration of Cys (ca. 10.0 equiv) afforded a quicker and more dramatic fluorescent response. The pseudofirst-order rate of the reaction is found to be 1.4 × 10^−2^·s^−1^ (Figure S7). And 1 h was set as the optimized reaction time as the fluorescence intensity reached a plateau within 1 h at each inspected concentration of Cys.

The effect of pH on the response of **1** toward Cys was studied. Without Cys, the fluorescence intensity of the probe remained unchanged with pH ≤8 and increased significantly with the pH value over 8, indicating that probe **1** is stable under the neutral condition and prone to hydrolysis under the alkaline condition (Figure 2(b)). With addition of Cys, the fluorescence was gradually increased in the region of pH 4.0–7.0 and reached the maximum at pH 7.4. These results demonstrated that **1** responds well to Cys at round physiological pH.

### 3.3. Sensitivity and Selectivity

**Figure 3:**
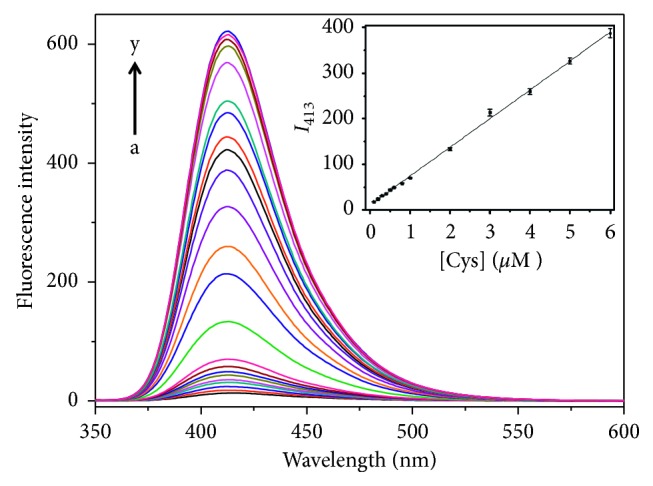
Fluorescence spectra of probe **1** (10 *μ*M) in the presence of Cys with various concentrations: 0, 0.1, 0.2, 0.3, 0.4, 0.5, 0.6, 0.8, 1.0, 2.0, 3.0, 4.0, 5.0, 6.0, 7.0, 8.0, 9.0, 10.0, 20.0, 30.0, 40.0, 50.0, and 100.0 *μ*M (from *a* to *y*). Inset shows the standard curve. *λ*_ex_ = 347 nm.

The quantitative response ability of probe **1** towards Cys was inspected via fluorescence titration. The fluorescence intensity gradually increases with increment of Cys contents and reaches a plateau with the Cys concentration up to 30 *μ*M (Figure 3). And there is a good linear correlation between emission intensity at 413 nm and the Cys concentration in the range of 0.1–6 *μ*M. Linear equation can be expressed as *I* = 10.81 + 63.16 × [Cys]/*μ*M (*R* ^2^ = 0.998). The detection limit was estimated to be 23 nM (3σ). The analytical performances of probe **1** were also compared with other reported fluorescent Cys probes using 2,4-dinitrobenzenesulfonyl ester (DNBS) as the recognition moiety [46–55] (Table S1).

**Figure 4:**
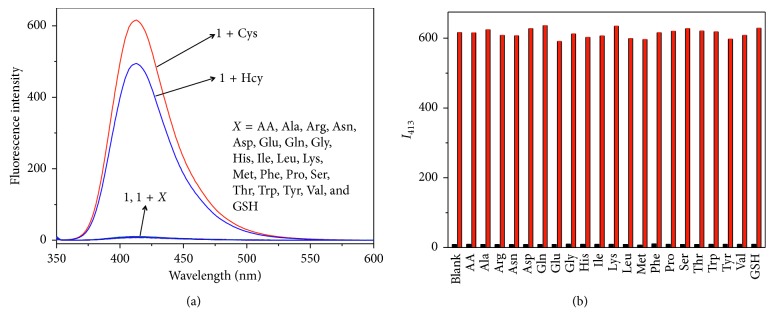
(a) Fluorescence spectra of probe **1** (10 *μ*M) towards different amino acids, AA and GSH (100 *μ*M). (b) Fluorescence intensities of probe **1** (10 *μ*M) at 413 nm upon the addition of different interfering species (100 *µ*M) (low bars), followed by addition of Cys (100 *μ*M) (high bars). *λ*_ex_ = 347 nm.

To evaluate the specificity of the assay, the spectral response of probe **1** towards various biologically relevant analytes including twenty natural protein amino acids, AA, Hcy, and GSH was recorded. Cys generated a significant enhancement of fluorescence intensity. Another biothiol, Hcy, also created an obvious fluorescent emission for the probe solution, indicating that probe **1** can response to both Cys and Hcy but showing a higher reactivity for Cys. Other analytes, including GSH, did not show any significant changes (Figure 4(a)). The higher reactivity of the probe toward Cys over GSH may be ascribed to the bulkiness of GSH and the significant steric hindrance around its thiol group. The spectral response of probe **1** for various metal ions (Al^3+^, Ca^2+^, Cd^2+^, C0^2+^, Cu^2+^, Fe^2+^, Fe^3+^, K^+^, Mg^2+^, Mn^2+^, Na^+^, Ni^2+^, Pb^2+^, and Zn^2+^) was also inspected (Figure S8), indicating that probe **1** is nonresponsive for these metal ions. Furthermore, competition experiments also indicated that the coexistence of other interfering species did not influence the reactivity of probe **1** for Cys (Figure 4(b)).

### 3.4. Sensing Mechanism

**Scheme 2:**
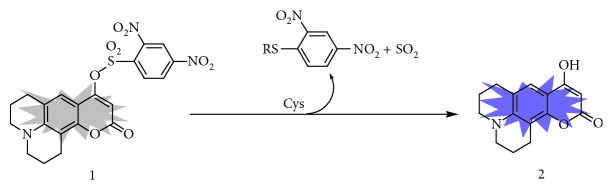
Sensing mechanism of probe **1** for Cys.

**Figure 5:**
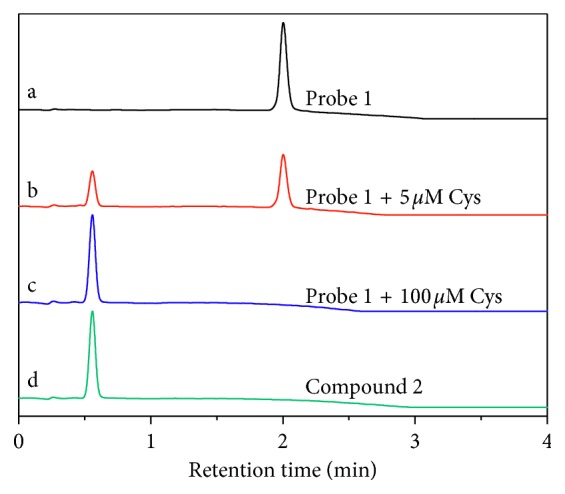
Reversed-phase HPLC chromatograms of (a) probe **1** (10 *μ*M), (b) probe **1** (10 *μ*M) and Cys (5 *μ*M), (c) probe **1** (10 *μ*M) and Cys (100 *μ*M), and (d) compound **2**.

The presented fluorescent Cys probe (**1**) was obtained by incorporating the DNBS functional group (a well-known recognition moiety for the biothiols) onto the coumarin-based fluorophore. Probe **1** is nonfluorescent due to the quenching effect of DNBS unit via the electron-transfer process. The introduced Cys can first react with DNBS of the probe via the nucleophilic aromatic substitution and form a unstable negative-charged intermediate, which further involved the intramolecular rearrangement to yield the sulfur dioxide, 2,4-dinitrophenyl cysteine, and compound **2** (a highly-emissive fluorophore) (Scheme 2). HPLC analysis was performed to verify this proposed sensing mechanism. **1** alone exhibited a single chromatographic peak at 2.00 min (curve a in Figure 5). After incubation probe **1** (10 *μ*M) with Cys (5 *μ*M), a new peak at 0.56 min appeared, which can be ascribed to compound **2** (curves b and d in Figure 5). Incubating probe **1** (10 *μ*M) with high concentration of Cys (100 *μ*M) resulted in the disappearance of the peak at 2.00 min and leaded to a chromatographic profile identical to that of compound **2**, which indicated that probe **1** can be completely converted to compound **2** upon the Cys-induced thiolysis process.

### 3.5. Cellular Imaging

**Figure 6:**
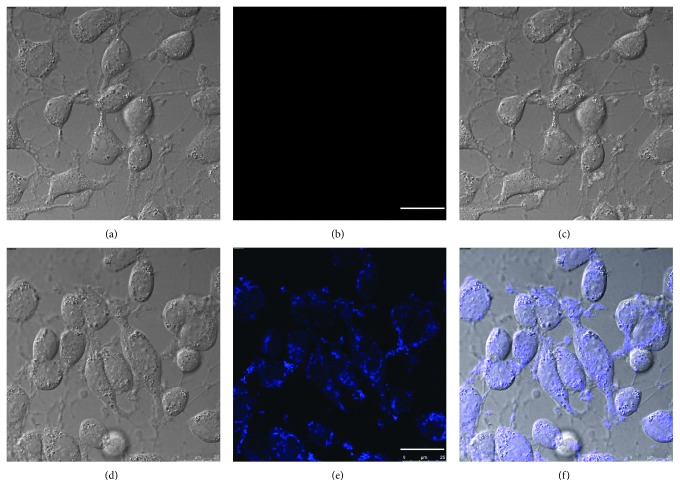
Confocal fluorescence images of living HeLa cells. Bright-field image (a) and fluorescence image (b) of cells incubated with probe **1** (10 *μ*M) for 1 h; (c) overlay of the images of (a) and (b); bright-field image (d) and fluorescence image (e) of cells incubated with probe **1** (10 *μ*M) for 1 h and subsequent treatment with Cys (100 *μ*M) for another 1 h; (f) overlay of the images of (d) and (e). *λ*_ex_ = 405 nm; scare bar = 25 *μ*m.

The good water solubility and high selectivity inspired us to use **1** for the bioimaging application. Firstly, cellular cytotoxicity of probe **1** was inspected (Figure S9). The high survival rates of all these three kinds of cells with different concentrations of probe **1** indicated that the probe was highly biocompatible. Then, cellular imaging experiments were conducted. HeLa cells incubated with probe **1** alone displayed no intracellular fluorescence (Figure 6(b)). However, cells incubated with **1** and consequently with Cys (100 *μ*M) exhibited strong blue fluorescent emission (Figure 6(e)). These imaging results indicated that probe **1** is living cell membrane permeable and can be used to monitor intracellular Cys.

## 4. Conclusions

In conclusion, we developed a turn-on fluorescent probe for Cys based on a coumarin-derived fluorophore. The sensing mechanism involved the Cys-induced cleavage of the DNBS group and the follow-up release of the coumarin fluorophore, which was confirmed by HPLC and spectral results. Probe **1** displayed high selectivity for Cys and a low detection limit of 23 nM. The proposed probe also features excellent water solubility and biocompatibility and has been successfully utilized for imaging Cys in living cells.

## Data Availability

The data used to support the findings of this study are available from the corresponding author upon request.

## Supplementary Materials

Supplementary Materials Supplementary Description Part 1: experimental materials, instrumentations, and experimental procedures for the HPLC test and cell viability assay/imaging. Part 2: characterization of compound **2** and probe **1**. Figure S1: 1H NMR chemical shifts of compound **2**. Figure S2: 13C NMR chemical shifts of compound 2. Figure S3: high-resolution mass spectrum (HRMS) of compound **2**. Figure S4: 1H NMR chemical shifts of probe **1**. Figure S5: 13C NMR chemical shifts of probe **1**. Figure S6: high-resolution mass spectrum (HRMS) of probe **1**. Part 3: kinetic study of **1** to Cys. Figure S7: the kinetic study of the response of probe **1** to Cys (10 equiv) under pseudofirst-order conditions based on the time course of the emission intensity at 413 nm. Part 4: comparison of DNBS-based fluorescent probes for Cys. Table S1: comparison of DNBS-based fluorescent probes for Cys. Part 5: spectral responses of probe **1** for various metal ions. Figure S8: fluorescence intensities of probe **1** (10 *μ*M) at 413 nm upon the addition of Cys (100 *µ*M) and different metal ions (100 *µ*M); *λ*_ex_ = 347 nm. Part 6: cell cytotoxicity of probe **1**. Figure S9: cell cytotoxicity of probe **1** against HeLa, A549, and MDA-MB-231 cells upon 24 h of incubation.
