# Supplementary material for: A Turn-On Fluorescent Probe for Sensitive Detection of Cysteine in a Fully Aqueous Environment and in Living Cells
Source: J Anal Methods Chem. 2018 Dec 13;2018:1986468. doi: 10.1155/2018/1986468 (PMC6311829; doi:10.1155/2018/1986468)
Supplement: Supplementary Materials — Supplementary Description Part 1: experimental materials, instrumentations, and experimental procedures for the HPLC test and cell viability assay/imaging. Part 2: characterization of compound 2 and probe 1. Figure S1: 1H NMR chemical shifts of compound 2. Figure S2: 13C NMR chemical shifts of compound 2. Figure S3: high-resolution mass spectrum (HRMS) of compound 2. Figure S4: 1H NMR chemical shifts of probe 1. Figure S5: 13C NMR chemical shifts of probe 1. Figure S6: high-resolution mass spectrum (HRMS) of probe 1. Part 3: kinetic study of 1 to Cys. Figure S7: the kinetic study of the response of probe 1 to Cys (10 equiv) under pseudofirst-order conditions based on the time course of the emission intensity at 413 nm. Part 4: comparison of DNBS-based fluorescent probes for Cys. Table S1: comparison of DNBS-based fluorescent probes for Cys. Part 5: spectral responses of probe 1 for various metal ions. Figure S8: fluorescence intensities of probe 1 (10 μM) at 413 nm upon the addition of Cys (100 µM) and different metal ions (100 µM); λ ex = 347 nm. Part 6: cell cytotoxicity of probe 1. Figure S9: cell cytotoxicity of probe 1 against HeLa, A549, and MDA-MB-231 cells upon 24 h of incubation. [file 1986468.f1.pdf]

Electronic Supplementary Information

A fully-aqueous turn-on fluorescent probe for sensitive detection of cysteine and in living cells

1. Experimental

1.1 Materials and instrumentations

All chemicals for synthesis were obtained from commercial suppliers and were used as received. Millipore water (18 MΩ cm^{-1}) was used to prepare all aqueous solutions. Absorption spectra were recorded using a Varian Cary 60 spectrophotometer (Agilent Technologies, Santa Clara, USA), and fluorescence spectra were recorded using a Varian Cary Eclipse fluorescence spectrophotometer (Agilent Technologies, Santa Clara, USA). NMR spectra were collected in $CDCl_3$ on a Bruker AVQ-400 spectrometer. Mass spectrometry was performed on a Waters Xevo G2-S QTof™ mass spectrometer (Waters, Milford, MA, USA).

1.2. HPLC tests

HPLC analysis was carried out on a Waters Acquity UPLC H-Class system (Milford, MA, USA) equipped with a quaternary solvent delivery system, a column oven, an auto sampler, and a photodiode array detector. The analytes were separated in gradient mode with a Waters ACQUITY BEH 2.1 × 50 mm C18 1.7 μm column. Flow rate was 0.5 mL/min. Eluent components were water (A) and acetonitrile (B). The mobile phase gradient was as follows: the proportion of phase B increased from 50 to 100% in 4.0 min. Detection wavelength was set at 380 nm.

1.3. Determination of the detection limit

The detection limit was calculated based on the fluorescence titration. Fluorescence emission spectrum of probe **1** was measured by ten timesand the standarddeviation (σ) of this blank measurement was achieved. The slope (k) was derived form the calibration curve for quantitative analysis of Cu^{2+}. The detection limit was determined

withthe following equation:

detection limit = 3σ/k

1.4. Cell viability assay and cell imaging

The toxicity of probe **1** towards living cells was determined by MTT (3-(4,5-dimethyl-2-thiazolyl)-2,5-diphenyl-2-H-tetrazolium bromide) assays. Three kinds of cell lines (HeLa, A549, and MDA-MB-231 cells) were evaluated. The cells were seeded in 96-well plates at a density of 5×10^3 cells per well and cultured for 24 h at 37 ^{o}C with 5% CO_2 in humidified environment. Then a series of different concentrations of probes (0, 5, 10, and 20 μM) were added to the separated wells, and the cells were incubated for an additional 24 h. MTT solution was then added into each well and then residual MTT solution was removed after 4 h. The MTT-formazan crystals were dissolved in 200 μL DMSO. The absorbance of each well was measured by a microplate reader (Bio-TekELx800) at the wavelength of 490 nm. The cell viability was assessed using the following Equation: Cell viability (%) = $A_T/A_0 \times 100\%$, where A_T is the absorbance of treated cells and A_0 is the control absorbance. Data of cell viability are given as mean ± standard deviation (S.D.) (three replicate measurements).

For cellular imaging experimets, the cultured Hela cells were incubated with probe **1** (10 μM), probe **1** (10 μM) and consequently with Cys (100 μM) in DMEM (Dulbecco's Modified Eagle Medium) at 37 ^{o}C, respectively. The incubation time was set at 1 h. After incubation for the corresponding time, the cells were washed with PBS three times to remove free compound and ions before analysis. Then the cells

were imaged on confocal microscope (Olympus, FV1000, Japan).

2. Characterization of compound 2 and probe 1

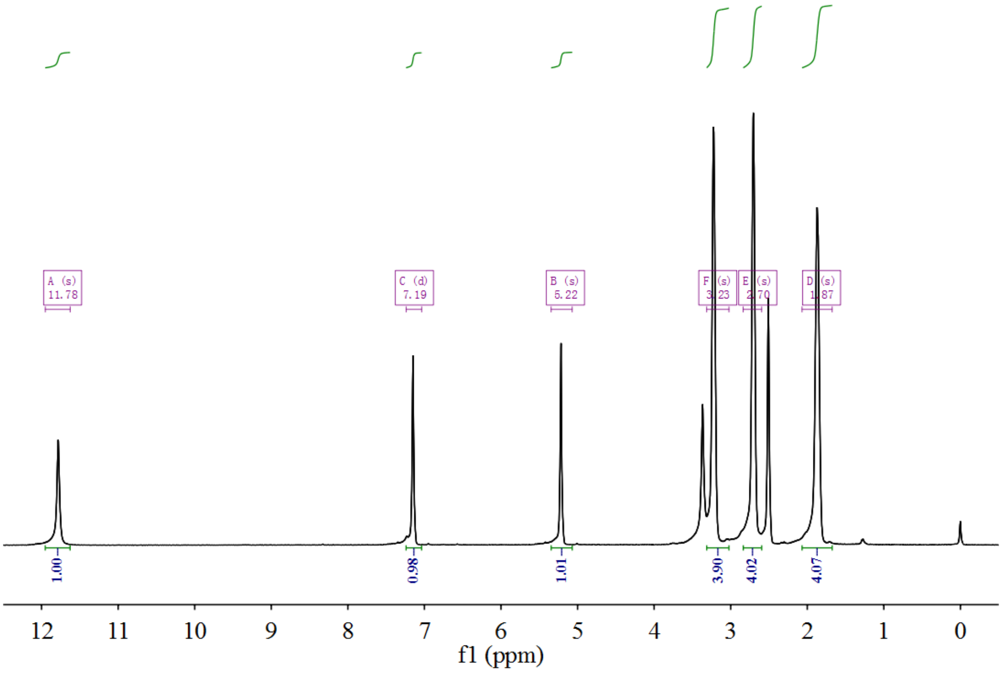

Figure S1. ^{1}H NMR chemical shifts of compound **2**.

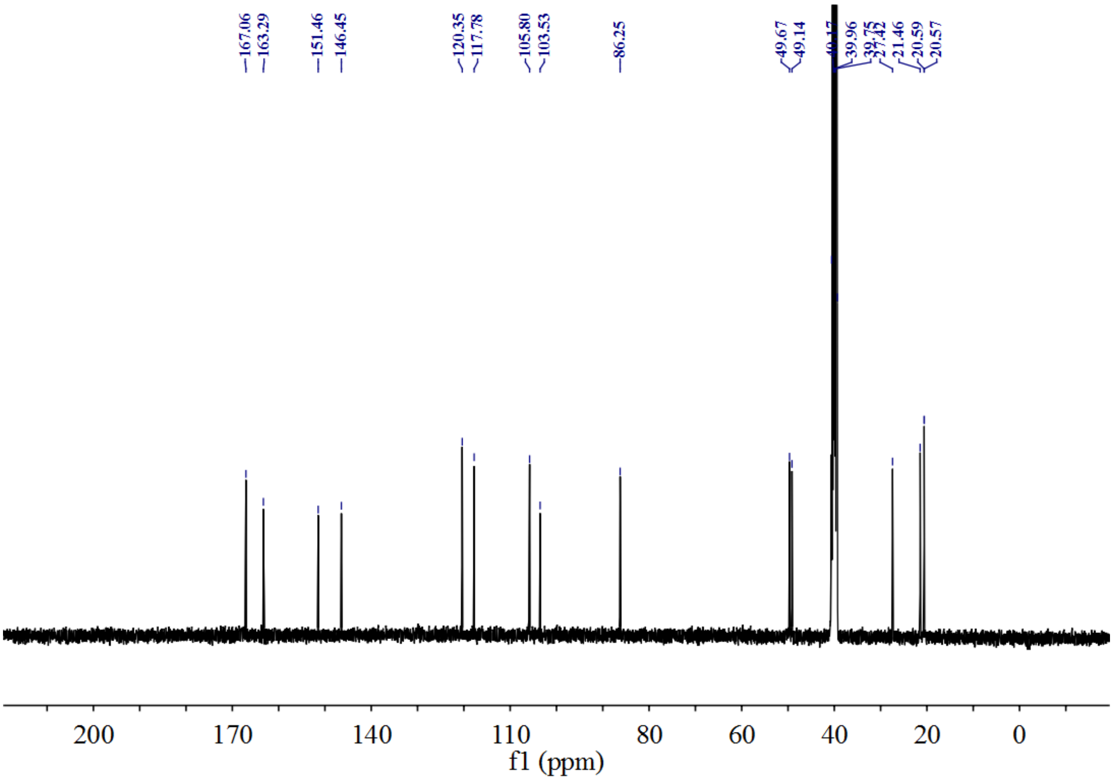

Figure S2 ^{13}C NMR chemical shifts of compound **2**.

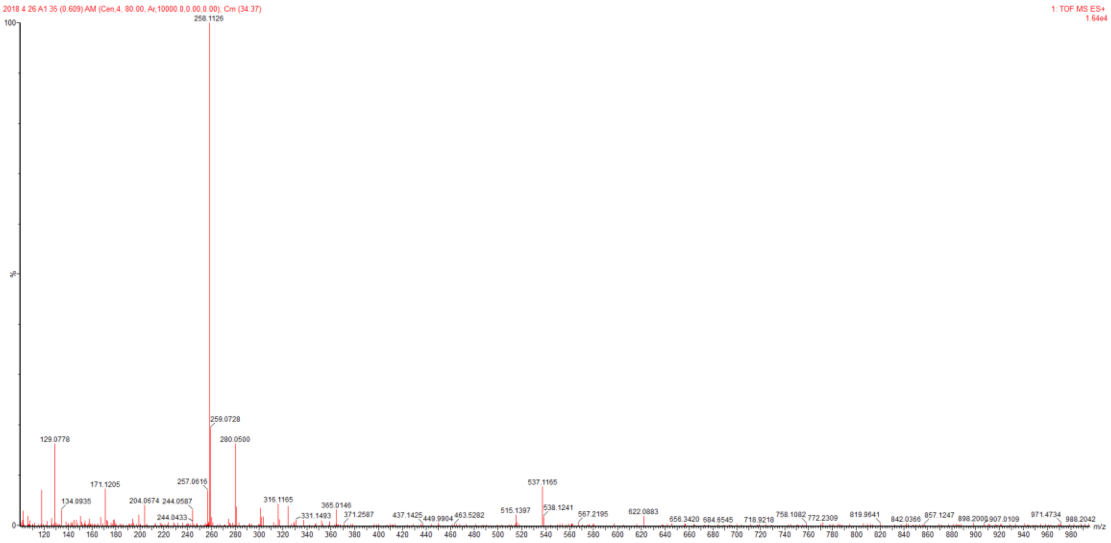

Figure S3 High resolution mass spectrum (HRMS) of the compound **2**.

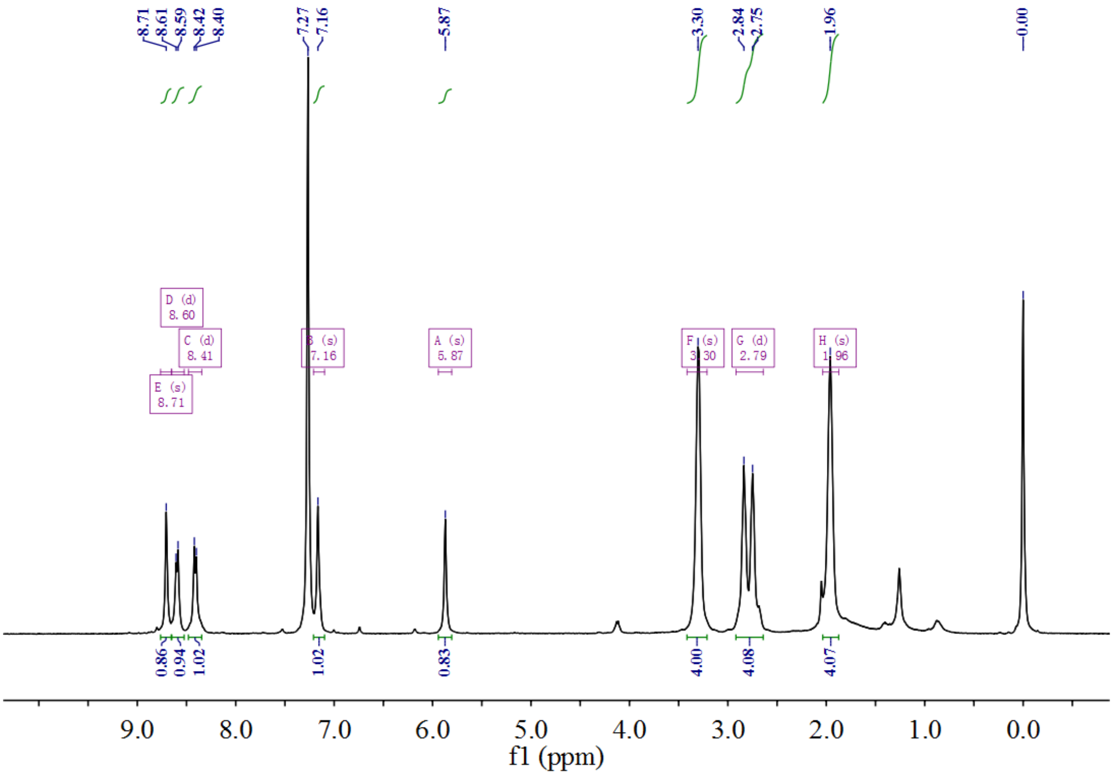

Figure S4. ^{1}H NMR chemical shifts of probe **1.**

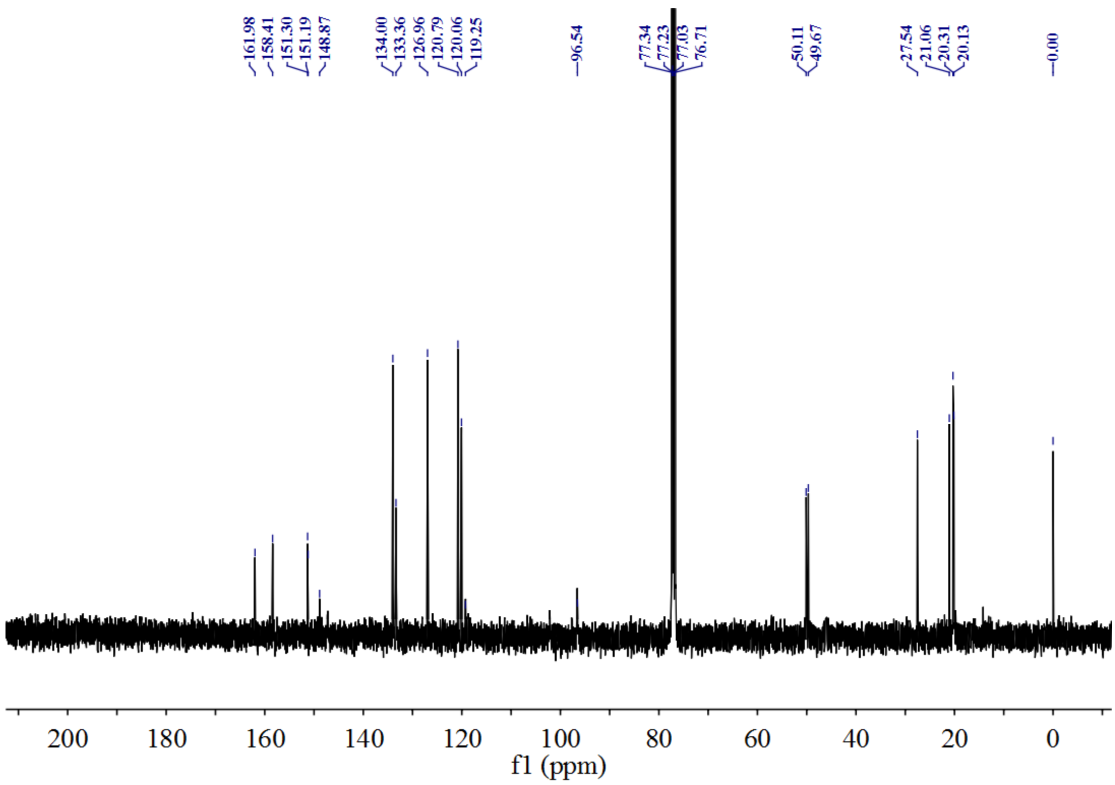

Figure S5 ^{13}C NMR chemical shifts of probe **1**.

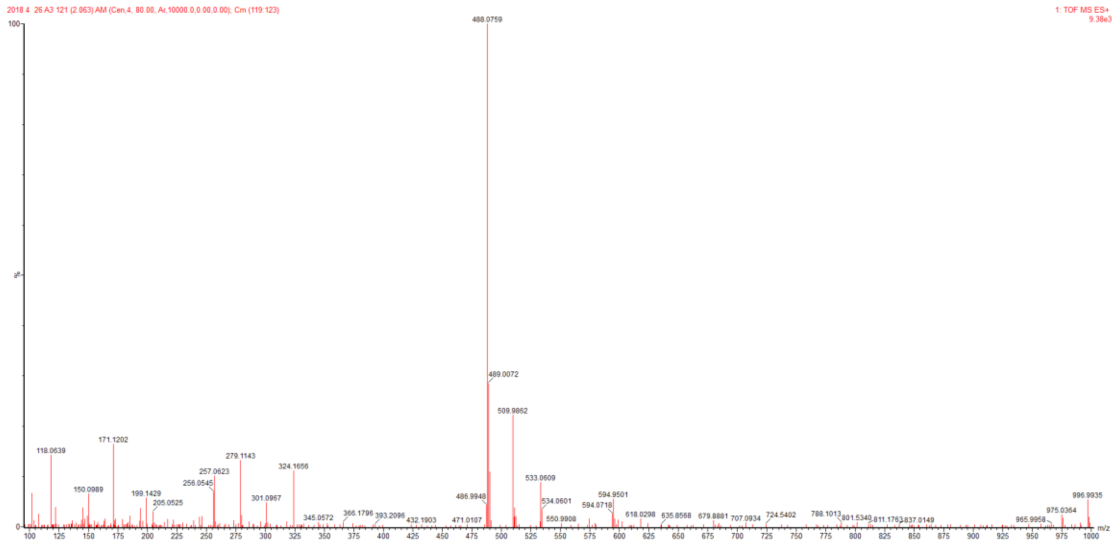

Figure S6 High resolution mass spectrum (HRMS) of the probe **1**.

3. Kinetic study of 1 to Cys

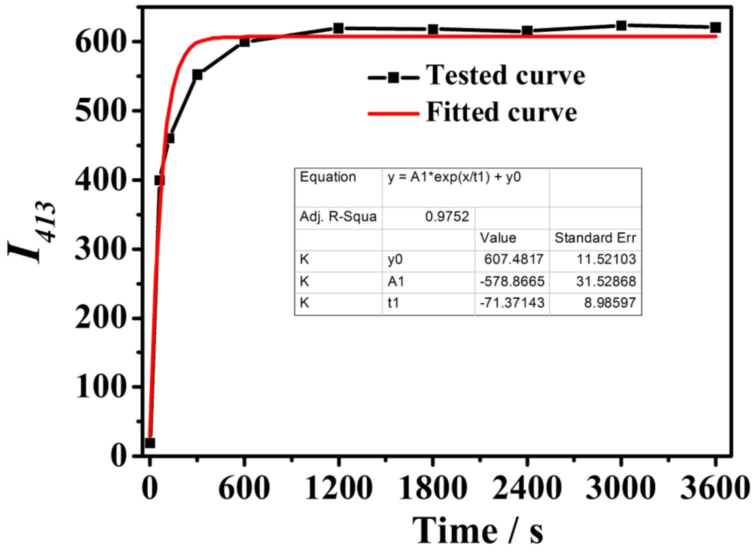

Figure S7 The kinetic study of the response of probe **1** to Cys (10 equiv) under pseudo-first-order conditions based on the time course of the emission intensity at 413 nm.

4. Comparison of DNBS-based fluorescent probes for Cys

Table S1. Comparison of DNBS -based fluorescent probes for Cys

Probes	$\lambda_{ex}/\lambda_{em}$ (nm)	Response mode	Media	LOD (μM)	Response time (min)	Real samples	Ref.
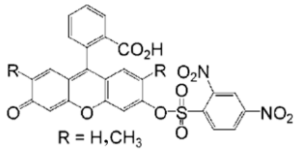	460/560	Turn on	EtOH/HEPES buffer (0.5/99.5, 10mM, 7.4)	2.0 pmol/ well	10	Enzyme assay	47
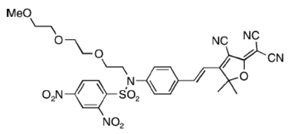	560/623	Turn on	HEPES buffer (10 mM, 7.4)	--	20	3T3 cells	48
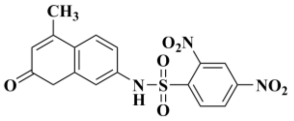	353/450	Turn on	Phosphate Buffer (10 mM, 9.0)	0.03	120	Hela cells	49
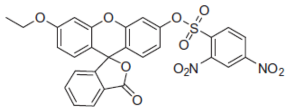	454/521	Turn on	DMSO/PBS (3/7, 10 mM, 7.4)	0.16	3	--	50

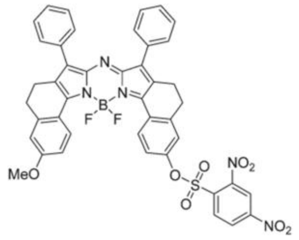	670/755	Turn on	MeCN/H_2O/DMSO (79/20/1, v/v/v; pH 7.5)	0.5	60	--	51
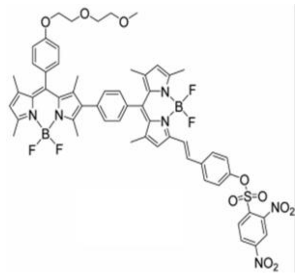	505/590	Turn on	MeOH/H_2O (4/1, v/v).	--	>100	SGC-H446 cells	52
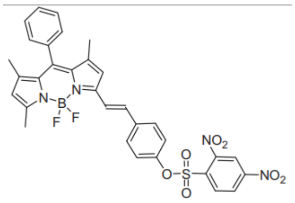	520/590	Turn on	MeOH/H_2O (4/1, v/v)	7.2	20	SGC-H446 cells	53
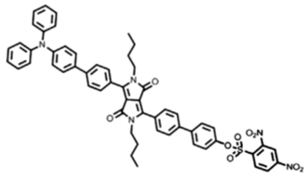	510/615	Turn on	DMSO/HEPES (7/3, v/v, 7.4)	5	30	Hela cells	54
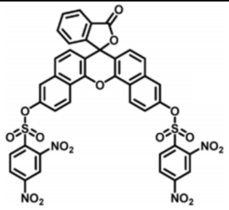	620/688	Turn on	DMSO/PBS (1/1, 10 mM, 7.4)	2.93	20	A549 cells	55
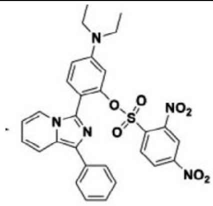	309/510	Turn on	ACN/PBS (1/4, 50 mM, 7.4)	0.17	10	A549 cells	56
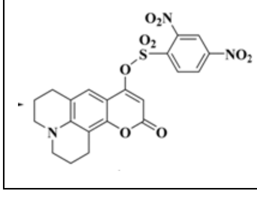	347/413	Turn on	PBS (10 mM, 7.4)	0.023	60	Hela cells	This work

5. Spectral responses of probe 1 for various metal ions

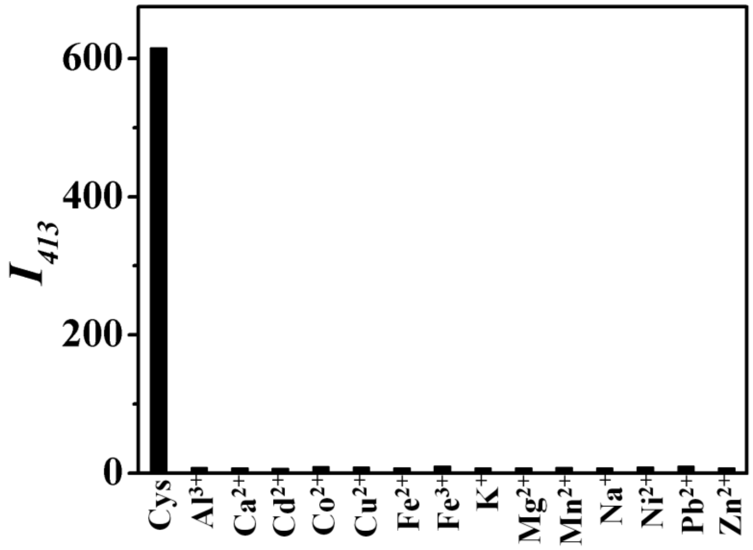

Figure S8 Fluorescence intensities of probe **1** (10 μM) at 413 nm upon the addition of Cys (100 μM) and different metal ions (100 μM). λex = 347 nm.

6. Cell cytotoxicity of probe 1

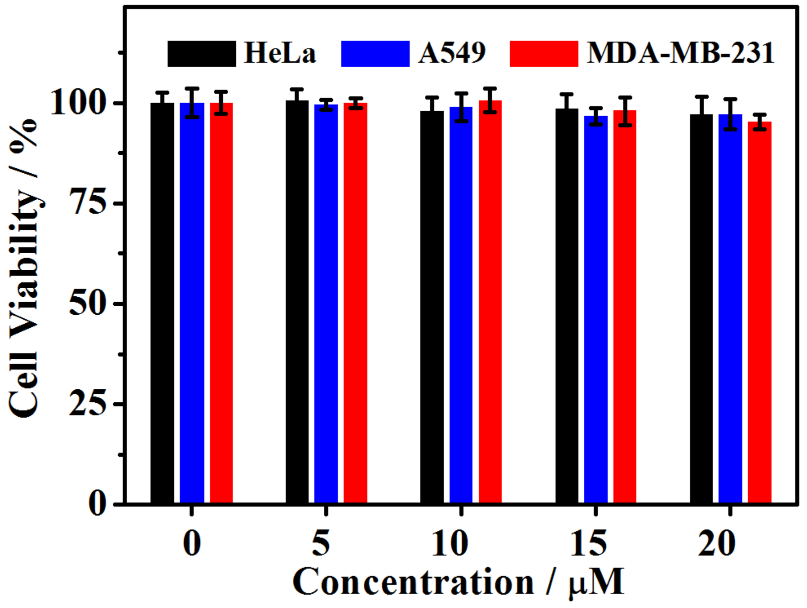

Figure S9 Cell cytotoxicity of probe **1** against HeLa, A549, and MDA-MB-231 cells upon 24 h of incubation.
